# Estimating the cost of cervical cancer screening in five developing countries

**DOI:** 10.1186/1478-7547-4-13

**Published:** 2006-08-03

**Authors:** Jeremy D Goldhaber-Fiebert, Sue J Goldie

**Affiliations:** 1 Program in Health Decision Science, Harvard School of Public Health, Harvard University, 718 Huntington Avenue, Boston, MA, 02115, USA

## Abstract

**Background:**

Cost-effectiveness analyses (CEAs) can provide useful information to policymakers concerned with the broad allocation of resources as well as to local decision makers choosing between different options for reducing the burden from a single disease. For the latter, it is important to use country-specific data when possible and to represent cost differences between countries that might make one strategy more or less attractive than another strategy locally. As part of a CEA of cervical cancer screening in five developing countries, we supplemented limited primary cost data by developing other estimation techniques for direct medical and non-medical costs associated with alternative screening approaches using one of three initial screening tests: simple visual screening, HPV DNA testing, and cervical cytology. Here, we report estimation methods and results for three cost areas in which data were lacking.

**Methods:**

To supplement direct medical costs, including staff, supplies, and equipment depreciation using country-specific data, we used alternative techniques to quantify cervical cytology and HPV DNA laboratory sample processing costs. We used a detailed quantity and price approach whose face validity was compared to an adaptation of a US laboratory estimation methodology. This methodology was also used to project annual sample processing capacities for each laboratory type. The cost of sample transport from the clinic to the laboratory was estimated using spatial models. A plausible range of the cost of patient time spent seeking and receiving screening was estimated using only formal sector employment and wages as well as using both formal and informal sector participation and country-specific minimum wages. Data sources included primary data from country-specific studies, international databases, international prices, and expert opinion. Costs were standardized to year 2000 international dollars using inflation adjustment and purchasing power parity.

**Results:**

Cervical cytology laboratory processing costs were I$1.57–3.37 using the quantity and price method compared to I$1.58–3.02 from the face validation method. HPV DNA processing costs were I$6.07–6.59. Rural laboratory transport costs for cytology were I$0.12–0.64 and I$0.14–0.74 for HPV DNA laboratories. Under assumptions of lower resource efficiency, these estimates increased to I$0.42–0.83 and I$0.54–1.06. Estimates of the value of an hour of patient time using only formal sector participation were I$0.07–4.16, increasing to I$0.30–4.80 when informal and unpaid labor was also included. The value of patient time for traveling, waiting, and attending a screening visit was I$0.68–17.74. With the total cost of screening for cytology and HPV DNA testing ranging from I$4.85–40.54 and I$11.30–48.77 respectively, the cost of the laboratory transport, processing, and patient time accounted for 26–66% and 33–65% of the total costs. From a payer perspective, laboratory transport and processing accounted for 18–48% and 25–60% of total direct medical costs of I$4.11–19.96 and I$10.57–28.18 respectively.

**Conclusion:**

Cost estimates of laboratory processing, sample transport, and patient time account for a significant proportion of total cervical cancer screening costs in five developing countries and provide important inputs for CEAs of alternative screening modalities.

## Background

Cervical cancer disproportionately affects women in developing countries [1]. Unlike most cancers, cervical cancer is preventable through cytologic screening programs that detect and treat precancerous lesions. In countries that have been able to achieve broad screening coverage at frequent intervals, mortality from cervical cancer has decreased considerably [2-7]. However, in the majority of low-income countries, cytologic screening has proven difficult to sustain, in large part because of its reliance on highly trained cytotechnologists, high-quality laboratories, and an infrastructure to support up to 3 visits for screening, colposcopic evaluation of abnormalities, and treatment.

Several factors have led to an expansion of the options for cervical cancer control. First, the availability of reliable HPV DNA assays has led to numerous studies documenting its higher sensitivity for detecting precancerous lesions compared with a single cytology test. Second, recent studies suggest that alternate screening strategies that use HPV DNA testing or simple visual screening methods may be more practical in some areas of the world [8-19]. Third, regardless of initial screening test (e.g., cervical cytology, HPV DNA testing, simple visual screening), strategies that enhance the linkage between screening and treatment, and seek to minimize loss to follow-up, have the best chance of measurable success [16,20]. Additionally, economic evaluations of these alternatives have concluded that they are promising [21-23].

Screening alternatives rely on different levels and types of resources such as laboratory infrastructure, staff mix, and clinical visits. These differences have important implications for the magnitude of the actual, total screening cost for each woman. Most importantly, they are not captured in the simple "assay cost" of each alternative – the staff time, supplies and equipment needed to collect a cervical sample. Furthermore, such differences can be magnified by country-specific characteristics such as population density, availability of staff and facilities, and topography. Therefore, cost-effectiveness results in one country may differ from those in another country.

We conducted a cost-effectiveness analysis of screening strategies in India, Kenya, Peru, South Africa, and Thailand [24]. For this analysis, it was necessarily to estimate the costs of delivering cervical cancer screening to a population of eligible women in each country. We estimated costs using resource quantities and prices actually experienced in these five countries when available, relying on expert opinion to standardize assumptions on resource quantities, useful life of equipment, and programmatic costs that could be realistically expected in national-level screening programs.

We identified three areas for which cost data were unavailable, and for which country-specific characteristics seemed particularly important to reflect. These included the cost of laboratory processing of cervical samples; the cost of transporting cervical samples from clinical sites to the laboratory; and the value of patient time traveling, waiting, and receiving care. We supplemented limited primary cost data in these three areas by developing alternative estimation methods. These estimation methods were then used in a CEA of alternative cervical cancer screening approaches based on three different initial screening tests: simple visualization methods, HPV DNA testing, and cervical cytology.

## Methods

To estimate the costs associated with screening, we adhered to the general guidelines recommended for performing cost-effectiveness analyses [25-28]. A societal perspective was adopted to estimate all costs associated with screening regardless of to whom each cost accrued. We included direct medical costs of screening, including staff, supplies, equipment, and facilities. We also included direct non-medical costs including patient time and transport involved in receiving care. In addition to estimates from a societal perspective, relevant costs were also estimated from a public health system payer perspective, focusing on laboratory transport and processing in relationship to the other direct medical costs involved in screening.

Cost estimates for three cervical cancer screening technologies – cervical cytology; HPV DNA testing with Hybrid Capture 2; and simple visual screening – were required (Table 1). However, because the focus of this analysis is largely laboratory transport and processing, we only provide laboratory-related cost estimates for cervical cytology and HPV DNA testing.

**Table 1:** Description of Screening Technologies

Screening Technology	Test Performance (1)	Description
Simple Visual Screening	Sensitivity: 67–79% Specificity: 49–86%	• Uses acetic acid to reveal acetowhite lesions • For abnormal results, some advocate use with immediate cryotherapy – "see and treat" in a single visit • Does not require special sample collection or laboratory processing equipment • Low level health personnel can be trained to perform • Personnel require supervision and retraining to maintain test performance • Quality Assurance/Quality Control difficult to assess • Generally requires 1–2 patient visits before treatment

Cervical Cytology	Sensitivity: 47–62% Specificity: 60–95%	• Cervical smear taken and then sample prepared on slides or in liquid media for transport • Because sample is generally examined in a laboratory, more than one patient visit may be required prior to treatment • Sample collection equipment is minimal, but some laboratory equipment required • Laboratory processing requires trained cytotechnicians and cytopathologists • Human evaluation of samples requires supervision and retraining to maintain test performance • Established Quality Assurance/Quality Control methods exist • Generally requires 3 patient visits before treatment

HPV DNA Testing with Hybrid Capture 2	Sensitivity: 66–100% Specificity: 61–96%	• Cervical sample taken and prepared for transport • Because sample is generally tested in a laboratory, more than one patient visit may be required prior to treatment • Sample collection kit and laboratory equipment required • Laboratory processing is automated requiring fewer personnel resources with less training • Results are quantitative in nature • Established Quality Assurance/Quality Control methods exist • Generally requires 2–3 patient visits before treatment

(1) Sankaranarayanan 2005

First, all activities associated with each screening technology were identified. Then, for each activity, we identified all resources used. Resources were categorized as either direct medical or direct non-medical. Direct medical resource inputs included staff time, disposable supplies, equipment and facilities depreciation used both for the collection of cervical samples as well as for the transport and laboratory processing of these samples. Direct non-medical resource inputs included patient transportation from home to the site where cervical samples were collected as well as patient time spent traveling, waiting, and interacting with medical staff.

Unit cost data for each resource type were compiled. Because these unit costs were derived from more than one year, country-specific deflators were used to adjust all costs to constant year 2000 terms [29]. Inflation adjustment was carried out prior to conversion of costs from local currency units to common currency units. To aid in cross-country comparability Purchasing Power Parity (PPP) exchange rates were used to convert costs expressed in year 2000 local currency units to year 2000 international dollars (I$) [27]. It was assumed that in the short term, equipment and supplies requiring complex manufacturing processes would be acquired on the international market and would be imported for use in a country's screening program, potentially as part of an international donor program.

The quantity of each resource was multiplied by its associated unit cost. These results were then summed to estimate the total cost of screening as well as the cost of each component.

### Data sources

Demonstration projects from five countries provided primary data on screening activities, resource categories, resource quantities, and unit costs. For staff costs, country-specific data from hospital and national salary scales for categories of health personnel were used.

An expert panel was provided with the primary cost and resource data from all countries and consulted to produce a standardized list of resource types and quantities that reflected the expected usage patterns for national screening programs [30]. Experts also provided the type and cost of laboratory equipment, the equipment's useful life, and level of productivity of laboratory staff for both cervical cytology and HPV DNA testing using Hybrid Capture 2.

We identified three areas for which primary data collected from in-country demonstration projects were more difficult to generalize. These included: (1) costs associated with laboratory processing of samples for either cervical cytology or HPV DNA testing using Hybrid Capture 2; (2) costs associated with transporting laboratory samples from the site of collection to the laboratory for processing; (3) costs of women's time traveling to and from the site of service delivery, waiting for service delivery, and receiving the service. For each of these areas, alternate estimation methods were employed.

### Laboratory sample processing

To estimate the cost of laboratory sample processing, we took a detailed quantity-and-price approach for cervical cytology and HPV DNA laboratory sample processing. Simple visual screening did not require samples from the initial screening visit to be processed in a laboratory, and thus no estimate of this type of laboratory processing costs was made for simple visual screening.

Staff requirements, productivity levels, and equipment depreciation were estimated by an expert panel who had significant experience with implementing cervical cancer screening and in developing country healthcare provided input on laboratory processing [30]. Staff costs were based on country-specific data from hospital and national salary scales. Supply and equipment costs were estimated using primary data in the five countries as well as international price data (Digene Corporation, Gaithersburg, MD, USA, 2000). For all equipment depreciation, we used straight-line depreciation discounting with a 3% discount rate and assumed no end-term resale value [28].

Because laboratory sample processing is relatively complex and certain elements such as facilities costs are difficult to estimate without detailed information from established laboratories in each country, we compared the detailed quantity-and-price approach to a previously published analysis of US-based cervical cytology laboratory costs. We modified the method used in the previously published analysis to provide comparative, country-specific estimates based on productivity levels for cytotechnicians and cytopathologists, simplified facilities costs, and a lump sum disposable supply cost for the five countries of interest [31]. To estimate facilities costs we compared the ratio of general per-meter facilities costs in each of the five countries with those of the US and used these ratios to form five multipliers [27]. Then, after adjusting the US-based analysis's detailed estimate of facilities costs for inflation, we used the five multipliers to estimate facilities costs in each of the five countries [31]. This method required wage rates for laboratory technicians and pathologists, productivity levels for technicians, expected abnormal sample rate and negative review, facilities requirements, and lump-sum supplies costs. Because productivity levels in this method were not assumed to differ between countries, cost variation was due to differences in the input costs necessary to achieve the target productivity level. Since the validation exercise makes specific productivity assumptions, it also allows for direct estimates of annual sample processing capacities for each type of laboratory.

Because cytology laboratories rely more heavily on human productivity than on automated processing equipment and HPV DNA laboratories rely more heavily on automated processing equipment than human productivity, we performed a sensitivity analysis on our estimates in which we varied the staff productivity assumptions from 33–200% for cytology laboratories and the equipment costs between 33–200% for HPV DNA laboratories.

### Laboratory sample transport

We used the laboratory sample processing capacity estimates for each type of laboratory as an input for estimating the cost of laboratory sample transport, with the exception of simple visual screening which does not routinely require laboratory processing of samples collected at the initial screening visit.

**Figure 1:**
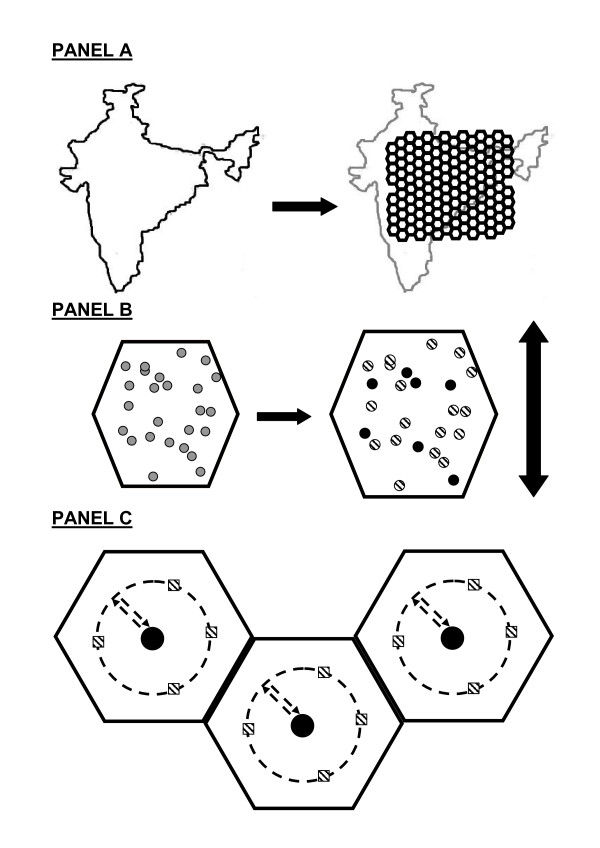
Spatial Model for Laboratory Sample Transport Cost Estimation*. Panel A shows the superimposition of a uniform grid of polygons onto a country's land area. Each polygon represents the rural area serviced by one laboratory unit. Panel B shows that the size of each polygon is not determined by rural population density (gray circles in left polygon) but rather by the density of screening eligible patients (black circles in right polygon). Panel C shows three laboratory areas each being serviced by a laboratory (black circle in center of each polygon). The driving route originates in the center following the dashed line to a circle with radius equal to half that of the polygon that visits each screening clinic site (dashed squares) before returning to the laboratory at the center. *The India outline map shown in the figure was made freely available from ; accessed: 7/22/2005

We used a spatial model to estimate transport costs. Based on a country's land area, population size, population structure, and percent rural population, we estimated the density of screen-eligible women. In this case, we wished to estimate the average, rural density of 35 year-old women because this was the target screening group for each year (Figure 1) [32,33]. We assumed that the rural population was uniformly and regularly distributed over the country's land area.

RuralDensityScreenEligibles=Population*EligibleAge%*Rural%LandArea
 MathType@MTEF@5@5@+=feaafiart1ev1aaatCvAUfKttLearuWrP9MDH5MBPbIqV92AaeXatLxBI9gBaebbnrfifHhDYfgasaacH8akY=wiFfYdH8Gipec8Eeeu0xXdbba9frFj0=OqFfea0dXdd9vqai=hGuQ8kuc9pgc9s8qqaq=dirpe0xb9q8qiLsFr0=vr0=vr0dc8meaabaqaciaacaGaaeqabaqabeGadaaakeaacqWGsbGucqWG1bqDcqWGYbGCcqWGHbqycqWGSbaBcqWGebarcqWGLbqzcqWGUbGBcqWGZbWCcqWGPbqAcqWG0baDcqWG5bqEcqWGtbWucqWGJbWycqWGYbGCcqWGLbqzcqWGLbqzcqWGUbGBcqWGfbqrcqWGSbaBcqWGPbqAcqWGNbWzcqWGPbqAcqWGIbGycqWGSbaBcqWGLbqzcqWGZbWCcqGH9aqpdaWcaaqaaiabdcfaqjabd+gaVjabdchaWjabdwha1jabdYgaSjabdggaHjabdsha0jabdMgaPjabd+gaVjabd6gaUjabcQcaQiabdweafjabdYgaSjabdMgaPjabdEgaNjabdMgaPjabdkgaIjabdYgaSjabdwgaLjabdgeabjabdEgaNjabdwgaLjabcwcaLiabcQcaQiabdkfasjabdwha1jabdkhaYjabdggaHjabdYgaSjabcwcaLaqaaiabdYeamjabdggaHjabd6gaUjabdsgaKjabdgeabjabdkhaYjabdwgaLjabdggaHbaaaaa@8215@

For a laboratory functioning at a particular capacity level, we then determined the size of the area serviced by the lab:

LabArea=AnnualSamples*Capacity%RuralDensityScreenEligibles
 MathType@MTEF@5@5@+=feaafiart1ev1aaatCvAUfKttLearuWrP9MDH5MBPbIqV92AaeXatLxBI9gBaebbnrfifHhDYfgasaacH8akY=wiFfYdH8Gipec8Eeeu0xXdbba9frFj0=OqFfea0dXdd9vqai=hGuQ8kuc9pgc9s8qqaq=dirpe0xb9q8qiLsFr0=vr0=vr0dc8meaabaqaciaacaGaaeqabaqabeGadaaakeaacqWGmbatcqWGHbqycqWGIbGycqWGbbqqcqWGYbGCcqWGLbqzcqWGHbqycqGH9aqpdaWcaaqaaiabdgeabjabd6gaUjabd6gaUjabdwha1jabdggaHjabdYgaSjabdofatjabdggaHjabd2gaTjabdchaWjabdYgaSjabdwgaLjabdohaZjabcQcaQiabdoeadjabdggaHjabdchaWjabdggaHjabdogaJjabdMgaPjabdsha0jabdMha5jabcwcaLaqaaiabdkfasjabdwha1jabdkhaYjabdggaHjabdYgaSjabdseaejabdwgaLjabd6gaUjabdohaZjabdMgaPjabdsha0jabdMha5jabdofatjabdogaJjabdkhaYjabdwgaLjabdwgaLjabd6gaUjabdweafjabdYgaSjabdMgaPjabdEgaNjabdMgaPjabdkgaIjabdYgaSjabdwgaLjabdohaZbaaaaa@785F@

Each laboratory is assumed to serve all eligible individuals within a laboratory area. All laboratory areas taken together form a Voronoi diagram of a country's land area with each laboratory at the center of a specific Voronoi cell [34]. Within each laboratory area, the driving path for laboratory transport is assumed to originate at the laboratory, travel away from the center until it reaches a distance of half the radius of the lab area, follow a circle of half the radius of the lab area to collect samples from primary clinics, and return to the laboratory in the center. The length of the path driven is then:

DrivingLength=(1+π)LabAreaπ
 MathType@MTEF@5@5@+=feaafiart1ev1aaatCvAUfKttLearuWrP9MDH5MBPbIqV92AaeXatLxBI9gBaebbnrfifHhDYfgasaacH8akY=wiFfYdH8Gipec8Eeeu0xXdbba9frFj0=OqFfea0dXdd9vqai=hGuQ8kuc9pgc9s8qqaq=dirpe0xb9q8qiLsFr0=vr0=vr0dc8meaabaqaciaacaGaaeqabaqabeGadaaakeaacqWGebarcqWGYbGCcqWGPbqAcqWG2bGDcqWGPbqAcqWGUbGBcqWGNbWzcqWGmbatcqWGLbqzcqWGUbGBcqWGNbWzcqWG0baDcqWGObaAcqGH9aqpdaqadiqaaiabigdaXiabgUcaRGGaciab=b8aWbGaayjkaiaawMcaamaakaaabaWaaSaaaeaacqWGmbatcqWGHbqycqWGIbGycqWGbbqqcqWGYbGCcqWGLbqzcqWGHbqyaeaacqWFapaCaaaaleqaaaaa@4EE4@

The time spent driving this route was estimated by using percentage paved and unpaved roads in each country as well as the average speed driven on paved and unpaved roads [35,36].

DrivingTime=DrivingLength(Paved%*SpeedPaved+Unpaved%*SpeedUnpaved)
 MathType@MTEF@5@5@+=feaafiart1ev1aaatCvAUfKttLearuWrP9MDH5MBPbIqV92AaeXatLxBI9gBaebbnrfifHhDYfgasaacH8akY=wiFfYdH8Gipec8Eeeu0xXdbba9frFj0=OqFfea0dXdd9vqai=hGuQ8kuc9pgc9s8qqaq=dirpe0xb9q8qiLsFr0=vr0=vr0dc8meaabaqaciaacaGaaeqabaqabeGadaaakeaacqWGebarcqWGYbGCcqWGPbqAcqWG2bGDcqWGPbqAcqWGUbGBcqWGNbWzcqWGubavcqWGPbqAcqWGTbqBcqWGLbqzcqGH9aqpdaWcaaqaaiabdseaejabdkhaYjabdMgaPjabdAha2jabdMgaPjabd6gaUjabdEgaNjabdYeamjabdwgaLjabd6gaUjabdEgaNjabdsha0jabdIgaObqaamaabmGabaGaemiuaaLaemyyaeMaemODayNaemyzauMaemizaqMaeiyjauIaeiOkaOIaem4uamLaemiCaaNaemyzauMaemyzauMaemizaqMaemiuaaLaemyyaeMaemODayNaemyzauMaemizaqMaey4kaSIaemyvauLaemOBa4MaemiCaaNaemyyaeMaemODayNaemyzauMaemizaqMaeiyjauIaeiOkaOIaem4uamLaemiCaaNaemyzauMaemyzauMaemizaqMaemyvauLaemOBa4MaemiCaaNaemyyaeMaemODayNaemyzauMaemizaqgacaGLOaGaayzkaaaaaaaa@8093@

Four costs were calculated for laboratory transport. Two were derived from the estimates.

DriverCostPerSample=Salary*DrivingTimeNumSamplesYear
 MathType@MTEF@5@5@+=feaafiart1ev1aaatCvAUfKttLearuWrP9MDH5MBPbIqV92AaeXatLxBI9gBaebbnrfifHhDYfgasaacH8akY=wiFfYdH8Gipec8Eeeu0xXdbba9frFj0=OqFfea0dXdd9vqai=hGuQ8kuc9pgc9s8qqaq=dirpe0xb9q8qiLsFr0=vr0=vr0dc8meaabaqaciaacaGaaeqabaqabeGadaaakeaacqWGebarcqWGYbGCcqWGPbqAcqWG2bGDcqWGLbqzcqWGYbGCcqWGdbWqcqWGVbWBcqWGZbWCcqWG0baDcqWGqbaucqWGLbqzcqWGYbGCcqWGtbWucqWGHbqycqWGTbqBcqWGWbaCcqWGSbaBcqWGLbqzcqGH9aqpdaWcaaqaaiabdofatjabdggaHjabdYgaSjabdggaHjabdkhaYjabdMha5jabcQcaQiabdseaejabdkhaYjabdMgaPjabdAha2jabdMgaPjabd6gaUjabdEgaNjabdsfaujabdMgaPjabd2gaTjabdwgaLbqaaiabd6eaojabdwha1jabd2gaTjabdofatjabdggaHjabd2gaTjabdchaWjabdYgaSjabdwgaLjabdohaZjabdMfazjabdwgaLjabdggaHjabdkhaYbaaaaa@7144@

GasolineCostPerSample=Gasoline*DrivingLength*TripsPerYearNumSamplesYear
 MathType@MTEF@5@5@+=feaafiart1ev1aaatCvAUfKttLearuWrP9MDH5MBPbIqV92AaeXatLxBI9gBaebbnrfifHhDYfgasaacH8akY=wiFfYdH8Gipec8Eeeu0xXdbba9frFj0=OqFfea0dXdd9vqai=hGuQ8kuc9pgc9s8qqaq=dirpe0xb9q8qiLsFr0=vr0=vr0dc8meaabaqaciaacaGaaeqabaqabeGadaaakeaacqWGhbWrcqWGHbqycqWGZbWCcqWGVbWBcqWGSbaBcqWGPbqAcqWGUbGBcqWGLbqzcqWGdbWqcqWGVbWBcqWGZbWCcqWG0baDcqWGqbaucqWGLbqzcqWGYbGCcqWGtbWucqWGHbqycqWGTbqBcqWGWbaCcqWGSbaBcqWGLbqzcqGH9aqpdaWcaaqaaiabdEeahjabdggaHjabdohaZjabdYgaSjabdMgaPjabd6gaUjabdwgaLjabcQcaQiabdseaejabdkhaYjabdMgaPjabdAha2jabdMgaPjabd6gaUjabdEgaNjabdYeamjabdwgaLjabd6gaUjabdEgaNjabdsha0jabdIgaOjabcQcaQiabdsfaujabdkhaYjabdMgaPjabdchaWjabdohaZjabdcfaqjabdwgaLjabdkhaYjabdMfazjabdwgaLjabdggaHjabdkhaYbqaaiabd6eaojabdwha1jabd2gaTjabdofatjabdggaHjabd2gaTjabdchaWjabdYgaSjabdwgaLjabdohaZjabdMfazjabdwgaLjabdggaHjabdkhaYbaaaaa@88AD@

Additionally, vehicle maintenance was based on WHO-CHOICE data, and straight line depreciation of the initial vehicle purchase price was performed over the useful life of the vehicle [27]. The proportion of the time that the vehicle was used for laboratory sample transport was estimated by dividing the time spent driving the transport route by the total time a vehicle was in use each week. Then, vehicle maintenance costs and vehicle depreciation were multiplied by this proportion to estimate the vehicle costs attributable to sample transport.

The cost estimate produced by this method reflects rural laboratory sample transport costs. To calculate national average laboratory sample transport cost, an urban sample transport cost was estimated by multiplying the rural transport cost an efficiency factor associated with much higher urban population density. Then, a weighted average of these two costs was taken to produce a national average transport cost estimate.

A plausible range for sample transport cost was based on estimating transport costs using two alternative efficiency assumptions. First, we generated a lower bound by assuming complete efficiency – only the portion of vehicle use; depreciation; driver time; and gasoline consumption that were attributable to sample transport were included in the estimate. Second, we generated an upper bound by assuming that each driver and vehicle would sit idle when not being used for sample transport, attributing the total cost of driver and vehicle to sample transport.

Because the location and number of sites from which laboratory samples would be collected on the driving route was uncertain, we re-estimated our plausible range estimates based on efficiently using all resources but using a driving length that was 4 times the original length estimated.

### Value of patient time

It is difficult to estimate the value of women's time in developing countries using conventional approaches (e.g., average wage rates scaled by employments rates [26]) because of high rates of female participation in unpaid and informal labor [37]. We valued the percentage of women's time spent in formal sector employment by country-specific average wage rates and used country-specific minimum wage rates as proxies to value time spent performing informal and unpaid labor [37-43].

Method 1:

*proportion*_formal _* *wagerate*_*formal*_

Method 2:

*proportion*_formal _* *wagerate*_*formal *_+ *proportion*_informal _* *wagerate*_min_

We used the two methods to form reasonable bounds for sensitivity analyses. The first method was used to form the lower bound because it does not value productive time not spent in the formal sector. The second method was used to form the upper bound because it assumes that all potentially productive time not used in the formal sector is used for informal or unpaid labor and further assumes that the value of these activities is equivalent to a minimum wage.

## Results

**Figure 2:**
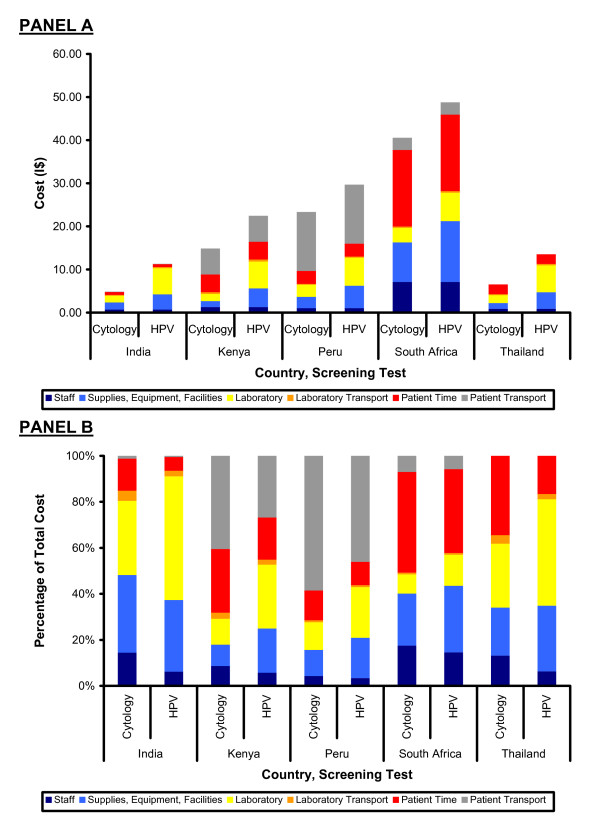
**Screening Cost Components (Totals and Proportions) from a Societal Perspective. **Panel A shows the component cost estimates for staff (dark blue), supplies, equipment, and facilities (light blue), laboratory processing (yellow), laboratory transport (orange), patient time (red), and patient transport (gray) for both cervical cytology and HPV DNA testing in India, Kenya, Peru, South Africa, and Thailand. Panel B shows these same cost components as proportions of the total cost.

**Figure 3:**
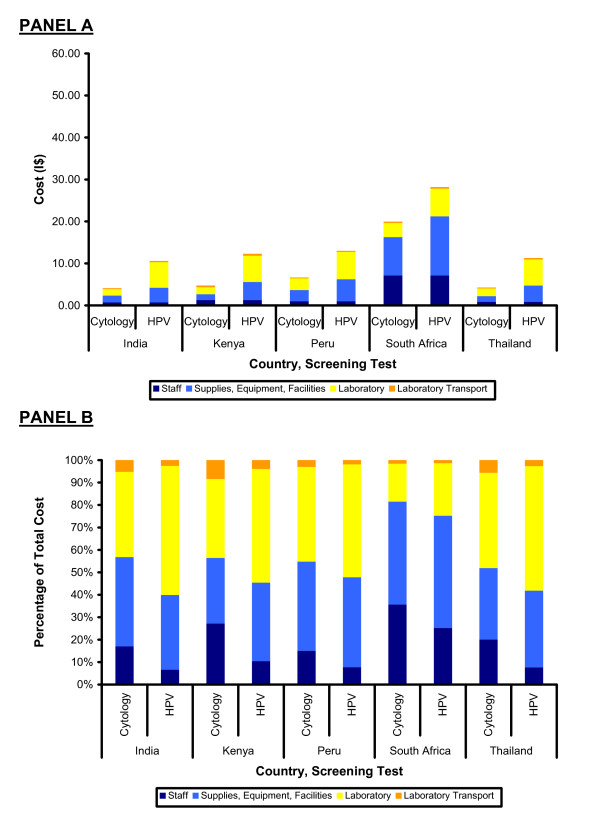
**Screening Cost Components (Totals and Proportions) from a Public Health System Payer Perspective**. Panel A shows the component cost estimates for staff (dark blue), supplies, equipment, and facilities (light blue), laboratory processing (yellow), laboratory transport (orange) for both cervical cytology and HPV DNA testing in India, Kenya, Peru, South Africa, and Thailand. Panel B shows these same cost components as proportions of the total cost.

A summary of the component costs making up the total cost of cervical cancer screening and their percentage contribution to the total is shown in Figure 2. Similar estimates from a public health system payer perspective (i.e, excluding patient time and transport) are shown in Figure 3. The direct medical costs of cervical cancer screening with cervical cytology excluding laboratory transport and laboratory sample processing were I$2.34, I$2.67, I$3.65, I$16.27, and I$2.21 for India, Kenya, Peru, South Africa, and Thailand respectively. With HPV DNA testing using Hybrid Capture 2, these costs were I$4.22, I$5.60, I$6.21, I$21.21, I$4.71. Based on primary data, expert opinion on quantity, productivity, and depreciation, and international prices, we produced detailed cost estimates of sample processing in cervical cytology laboratories and in HPV DNA laboratories that illustrate the relative contributions of component costs. Table 2 shows staff, supply, and equipment quantity, price, and depreciation data as well as the resulting cost estimates. Cervical cytology is more labor intensive, requiring a broader range and quantity of labor inputs with less reliance on equipment. HPV DNA laboratories rely on automated processing thus requiring less staff, although requiring specific equipment. Because of the uncertainty inherent in these estimates, Table 2 also shows the effect on laboratory processing costs when staff productivity assumptions are varied from 33% to 200% for cytology laboratories, and the equipment costs are varied from 33% to 200% for HPV DNA laboratories.

**Table 2:** Estimates of Laboratory Resources, Productivity Levels, and Costs

	India	Kenya	Peru	South Africa	Thailand	Source
Cervical Cytology Laboratory						

Staff						
Secretary (samples/week)	600	600	600	600	600	(1)
Secretary Wage (I$/hr)	1.48	2.31	2.57	4.06	2.29	(2)
Stainer (samples/week)	1,200	1,200	1,200	1,200	1,200	(1)
Stainer Wage (I$/hr)	1.48	2.31	2.57	4.06	2.29	(2)
Prep Tech (samples/week)	400	400	400	400	400	(1)
Prep Tech Wage (I$/hr)	1.65	n/a	2.57	4.29	2.29	(2)
Cytotechnician (samples/week)	200	200	200	200	200	(1)
Cytotechnician Wage (I$/hr)	2.47	n/a	5.65	5.71	3.48	(2)
Senior Cytotechnologist (samples/week)	1,200	1,200	1,200	1,200	1,200	(1)
Senior Cytotechnologist Wage (I$/hr)	4.94	5.39	12.52	14.24	4.25	(2)
Cytopathologist (samples/week)	1,200	1,200	1,200	1,200	1,200	(1)
Cytopathologist Wage (I$/hr)	8.23	11.55	12.92	18.09	5.97	(2)
Equipment						
Microscope (I$)	7,000	7,000	7,000	7,000	7,000	(1)
Microscopy Annuity Factor	2.8286	2.8286	2.8286	2.8286	2.8286	(1)

**Cost Estimate**						

Staff Costs (I$)	1.25	1.35	2.49	3.05	1.49	
Equipment/Supplies Costs (I$)	0.32	0.32	0.32	0.32	0.32	

**Total Cost**	**1.57**	**1.67**	**2.81**	**3.37**	**1.81**	
**Total Cost (productivity 33%, equipment 100%)**	**2.82**	**3.02**	**5.30**	**6.42**	**3.30**	
**Total Cost (productivity 200%, equipment 100%)**	**0.73**	**0.76**	**1.14**	**1.32**	**0.81**	

						

HPV DNA Laboratory						

Staff						
Secretary (samples/week)	450	450	450	450	450	(1)
Secretary Wage (I$/hr)	1.48	2.31	2.57	4.06	2.29	(2)
Lab Tech (samples/week)	450	450	450	450	450	(1)
Lab Tech Wage (I$/hr)	2.14	2.77	5.65	4.32	3.48	(2)
Pathologist (samples/week)	4,500	4,500	4,500	4,500	4,500	(1)
Pathologist Wage (I$/hr)	8.23	11.55	12.92	18.09	5.97	(2)
Supplies						
HPV Kit (I$)	5.33	5.33	5.33	5.33	5.33	(3)
Equipment						
HPV Equipment (including Microplate Luminometer) (I$)	35,000	35,000	35,000	35,000	35,000	(1)
HPV Equipment annuity factor	4.5797	4.5797	4.5797	4.5797	4.5797	(1)
Pipette Tips/Multichannel racks (I$)	1,800	1,800	1,800	1,800	1,800	(1)
Pipette Tips/Multichannel racks annuity factor	2.8286	2.8286	2.8286	2.8286	2.8286	(1)

**Cost Estimate**						

Staff Costs (I$)	0.39	0.55	0.85	0.91	0.57	
Equipment/Supplies Costs (I$)	5.68	5.68	5.68	5.68	5.68	

**Total Cost**	**6.07**	**6.23**	**6.53**	**6.59**	**6.25**	
**Total Cost (productivity 100%, equipment 200%)**	**11.76**	**11.92**	**12.22**	**12.28**	**11.94**	
**Total Cost (productivity 100%, equipment 33%)**	**2.27**	**2.43**	**2.73**	**2.79**	**2.45**	

(1) Expert Panel standardization assumptions; (2) Primary country-specific data from national pay scales and demonstration projects; (3) International price for public sector developing countries from Digene Corporation

Table 3 shows the results of the validation exercise we conducted based on a cytology laboratory cost analysis for the United States. The total costs estimated are similar to those in Table 2. The method used in the validation exercise requires fewer assumptions about staff inputs and productivity, does not specifically detail equipment and depreciation, and includes facility estimates. This approach was also used to estimate annual laboratory sample processing capacity based on technician productivity levels. For cervical cytology laboratories, we estimate a capacity of 28,800 samples processed per year for a laboratory unit of 6 cytotechnicians and 1 cytopathologist. For HPV DNA laboratories, we estimate a capacity of 21,600 samples processed per year for a laboratory of 1 technician and 1 pathologist.

**Table 3:** Estimate Validation: Laboratory Resources, Productivity Levels, and Costs

	India	Kenya	Peru	South Africa	Thailand	Sources
Cytology Laboratory Inputs						
Cytotechnician Salary (I$/hr)	2.47	3.48	5.65	5.71	3.48	(1)
Cytopathologist Salary (I$/hr)	8.23	11.55	12.92	18.09	5.97	(1)
Cytotechnician slides per hour	2.5	2.5	2.5	2.5	2.5	(2)
Cytopathologist slide review time (hr)	0.067	0.067	0.067	0.067	0.067	(2)
Abnormal slide (%)	0.120	0.120	0.120	0.120	0.120	(2)
Normal slides reviewed (%)	0.100	0.100	0.100	0.100	0.100	(2)
Square meter per laboratory	77.78	77.78	77.78	77.78	77.78	(2)
Facility cost per square meter ratio with US cost as base	0.057	0.025	0.091	0.041	0.030	(3)
Facility cost per year (I$)	6,735	2,925	10,765	4,875	3,540	
Samples processed per year	28,800	28,800	28,800	28,800	28,800	
Cost of supplies (I$)	0.25	0.25	0.25	0.25	0.25	(2)

**Cost Estimate**						

Staff Costs	1.10	1.55	2.40	2.53	1.47	
Facilities, Equipment, and Supplies Costs	0.48	0.35	0.62	0.42	0.37	

**Total Cost**	**1.58**	**1.90**	**3.02**	**2.95**	**1.84**	

(1) Primary country-specific data from national pay scales and demonstration projects; (2) Expert Panel standardization assumptions and Bishop's US cytology estimation method; (3) Expert Panel standardization assumptions, WHO-CHOICE data, and Bishop's US cytology estimation method

Table 4 shows the input parameters used to calculate the component costs of transporting laboratory samples from the clinical collection site to the laboratory for analysis. All parameters are derived from internationally available data sources for a broad set of countries. Table 5 shows that rural, per-sample transport costs vary from I$0.14–0.74 for HPV DNA laboratories and from I$0.12–0.64 from cervical cytology laboratories. Even though the areas served by cervical cytology laboratories are larger than the areas served by HPV DNA laboratories due to higher sample processing capacity, the cost per sample is lower because transports costs scale sub-linearly. The base case represents our lower bound of sample transport costs because it assumes efficiency of resource use for both driver and vehicle. If, however, all laboratory transport resources could not be used for other purposes when not being used for cervical cytology laboratory transport, the estimates would be between I$0.54–1.06 for HPV DNA laboratory transport and between I$0.42–0.83 for cytology laboratory transport. If the route the driver must take was increased four-fold to reflect both sparse road networks and dispersed screening sites, the estimates for cytology laboratory transport range from I$0.48–2.55 or I$0.68–2.78, depending on efficiency assumptions. For HPV DNA laboratory transport, the estimates range from I$0.55–2.95 or I$0.84–3.51, depending on efficiency assumptions.

**Table 4:** Rural Laboratory Sample Transport Parameters

	India	Kenya	Peru	South Africa	Thailand	Sources
Total Population	1,002,708,291	30,310,235	27,012,899	42,351,345	62,352,043	(1)
Women 35–39	32,872,209	660,717	867,291	1,420,154	2,557,418	(2)
Percent of Women Age 35	0.656	0.436	0.642	0.671	0.820	
Land Area (sq km)	2,973,190	569,140	1,280,000	1,221,040	510,890	(1)
Rural Population (% of total)	72.343	66.631	27.232	43.132	80.171	(1)
Roads, total network (km)	3,319,644	63,941.5	72,900	362,099	64,600	(1)
Roads, paved (% of total roads)	45.7	12.1	12.8	20.3	97.5	(1)
Annual HPV DNA Samples processed by HPV Lab equivalent per year	21,600	21,600	21,600	21,600	21,600	
Samples processed by Cytology Lab equivalent per year	28,800	28,800	28,800	28,800	28,800	
Average speed on Paved Road (km/hr)	90	90	90	90	90	(3)
Average speed on Unpaved Road (km/hr)	45	45	45	45	45	(3)
Driver Yearly Salary (I$)	6,675.33	10,661.03	2,665.93	10,661.03	3,881.02	(4)
Work Hours Per Year	2,300	2,400	2,400	2,100	2,500	(4)
Gasoline Cost per km (I$)	0.12	0.12	0.1	0.12	0.11	(4)
Monthly Maintenance (I$)	250.65	250.65	250.65	250.65	250.65	(4)
Cost of Vehicle (I$)	19,935.33	19,935.33	19,935.33	19,935.33	19,935.33	(4)
Depreciation Annuity Factor	7.7861	7.7861	7.7861	7.7861	7.7861	(4)

Cervical Cytology Laboratory						

Density of Screen Eligible Women (per sq km)	1.600	0.155	0.037	0.100	0.802	
Lab Area (sq km)	17,994.517	186,148.471	780,578.700	286,901.656	35,895.338	
Driving Length (km)	313.446	1,008.143	2,064.433	1,251.581	442.702	
Driving Time (hrs)	5.374	21.048	42.940	24.990	5.042	

HPV DNA Laboratory						

Density of Screen Eligible Women (per sq km)	1.600	0.155	0.037	0.100	0.802	
Lab Area (sq km)	13,495.888	13,9611.353	585,434.025	215,176.242	26,921.504	
Driving Length (km)	271.452	873.078	1,787.851	1,083.901	383.391	
Driving Time (hrs)	4.654	18.228	37.187	21.642	4.366	

(1) World Bank's World Development Indicators; (2) US Census Bureau's International Data Base – country-specific estimates; (3) International Center for Tropical Agriculture and World Bank; (4) WHO-CHOICE

**Table 5:** Rural Laboratory Transport Costs: Base Case and Efficiency Sensitivity Analyses

	India	Kenya	Peru	South Africa	Thailand
Resources Used Efficiently					
Cervical Cytology Laboratory					
Driver Cost	0.03	0.17	0.09	0.23	0.01
Gasoline Cost	0.07	0.22	0.37	0.27	0.09
Maintenance Cost	0.01	0.05	0.10	0.06	0.01
Depreciation Cost	0.01	0.04	0.08	0.06	0.01

**Total Cost**	**0.12**	**0.48**	**0.64**	**0.62**	**0.12**

HPV DNA Laboratory					
Driver Cost	0.03	0.19	0.10	0.26	0.02
Gasoline Cost	0.08	0.25	0.43	0.31	0.10
Maintenance Cost	0.01	0.05	0.11	0.07	0.01
Depreciation Cost	0.01	0.05	0.10	0.06	0.01

**Total Cost**	**0.14**	**0.55**	**0.74**	**0.72**	**0.14**

Resources Used in Whole Quantities					
Cervical Cytology Laboratory					
Driver Cost	0.23	0.37	0.09	0.37	0.13
Gasoline Cost	0.07	0.22	0.37	0.27	0.09
Maintenance Cost	0.10	0.10	0.10	0.10	0.10
Depreciation Cost	0.09	0.09	0.09	0.09	0.09

**Total Cost**	**0.49**	**0.78**	**0.66**	**0.83**	**0.42**

HPV DNA Laboratory					
Driver Cost	0.31	0.49	0.12	0.49	0.18
Gasoline Cost	0.08	0.25	0.43	0.31	0.10
Maintenance Cost	0.14	0.14	0.14	0.14	0.14
Depreciation Cost	0.12	0.12	0.12	0.12	0.12

**Total Cost**	**0.65**	**1.00**	**0.81**	**1.06**	**0.54**

Road Distance Quadrupled Resources Used Efficiently					
Cervical Cytology Laboratory					
Driver Cost	0.11	0.68	0.34	0.92	0.06
Gasoline Cost	0.27	0.87	1.49	1.08	0.35
Maintenance Cost	0.05	0.19	0.39	0.26	0.04
Depreciation Cost	0.04	0.16	0.33	0.22	0.04

**Total Cost**	**0.48**	**1.90**	**2.55**	**2.48**	**0.49**

HPV DNA Laboratory					
Driver Cost	0.13	0.78	0.40	1.06	0.07
Gasoline Cost	0.31	1.01	1.72	1.25	0.41
Maintenance Cost	0.06	0.22	0.45	0.30	0.05
Depreciation Cost	0.05	0.19	0.38	0.25	0.04

**Total Cost**	**0.55**	**2.20**	**2.95**	**2.86**	**0.57**

Road Distance Quadrupled Resources Used In Whole Quantities					
Cervical Cytology Laboratory					
Driver Cost	0.23	0.74	0.37	1.11	0.13
Gasoline Cost	0.27	0.87	1.49	1.08	0.35
Maintenance Cost	0.10	0.21	0.42	0.31	0.10
Depreciation Cost	0.09	0.18	0.36	0.27	0.09

**Total Cost**	**0.70**	**2.00**	**2.63**	**2.78**	**0.68**

HPV DNA Laboratory					
Driver Cost	0.31	0.99	0.49	1.48	0.18
Gasoline Cost	0.31	1.01	1.72	1.25	0.41
Maintenance Cost	0.14	0.28	0.56	0.42	0.14
Depreciation Cost	0.12	0.24	0.47	0.36	0.12

**Total Cost**	**0.88**	**2.51**	**3.25**	**3.51**	**0.84**

Estimates of patient time value using only formal sector wages and participation levels as well as those using weighted averages of formal sector wages and minimum wages are shown in Table 6. In countries such as India, Kenya, and Peru, where formal sector participation by women is low, the differences in estimated patient time value between the two methods is greater than 50%.

**Table 6:** Patient Time Costs in Economies with High Informal Sector Employment

	India	Kenya	Peru	South Africa	Thailand	Sources
Cost Inputs						

Average Formal Sector Wage Rates (I$/hr)	0.48	1.94	2.26	9.90	2.59	(1)
Women's Formal Employment As Percentage of Women's Non-Agricultural Employment	0.14	0.17	0.42	0.42	0.46	(2)
Average Minimum Wage Rate (I$/hr)	0.27	0.52	1.49	1.10	1.16	(3)

**Average Hourly Time Value**						

Formal Sector Only Patient Time Value (I$/hr)	**0.07**	**0.33**	**0.95**	**4.16**	**1.19**	
Weighted Average Patient Time Value (I$/hr)	**0.30**	**0.76**	**1.81**	**4.80**	**1.82**	

**Value of Time Traveling to, Waiting for, and Attending Cervical Cancer Screening**						

1-way Travel Time (mins)	30	110	30	48	15	(4)
Wait Time (health clinic) (mins)	60	90	25	111	30	(4)
Appointment Time (mins)	15	15	15	15	15	(5)

**Total Cost (Formal Sector Only) (I$)**	**0.16**	**1.79**	**1.58**	**15.39**	**1.49**	
**Total Cost (Formal and Informal Sector) (I$)**	**0.68**	**4.12**	**3.02**	**17.76**	**2.28**	

(1) US Department of Commerce country-specific estimates; (2) International Labour Organization; (3) US Department of Labor country-specific estimates, SalaryExpert, World Bank's World Development Indicators; (4) Primary country-specific data from demonstration projects; (5) Expert Panel standardization assumptions

## Discussion

The cost of laboratory processing, laboratory sample transport, and patient time accounted for 51%, 42%, 26%, 53%, and 66% of the total direct medical and non-medical costs of cervical cytology for India, Kenya, Peru, South Africa, and Thailand. For HPV DNA testing using Hybrid Capture 2, these percentages were 62%, 48%, 33%, 51%, and 65%. From a public health system payer perspective, with no patient time or patient transport costs included, laboratory processing and sample transport were 43%, 44%, 45%, 18%, and 48% of total direct medical costs for cervical cytology and 60%, 55%, 52%, 25%, and 58% of total direct medical costs for HPV DNA testing using Hybrid Capture 2 respectively.

The estimates presented in this paper differ slightly from those used in our previous paper primarily because we have updated and expanded the estimation methods and sensitivity analyses used to consider cervical cancer screening costs [24].

Cost-effectiveness analyses (CEAs) are increasingly used to assess the value provided by health care interventions for a given level of spending. Yet, it is difficult to evaluate the cost-effectiveness of delivering services that have not been previously implemented within a country, in part because real-world cost data on program implementation is lacking. Using only selected direct medical costs for which data is available – the "assay cost" – may lead to invalid cost estimates that exclude potentially important components. From a societal perspective, we believe that the three additional costs components estimated in this analysis account for between 26% and 66% of the per-patient cost of cervical cancer screening visits in India, Kenya, Peru, South Africa and Thailand. By varying assumptions in the estimation techniques it was possible to generate plausible ranges of costs useful for sensitivity analyses.

The quantity and price approach for estimating the cost of cervical cytology laboratory sample processing was generally consistent with estimates from our face validity exercise. The methods differed in two important ways. First, the quantity and price approach did not have sufficient data to estimate actual facilities costs associated with laboratory activity, whereas the method used for the validation exercise uses the average facilities cost within a given country as a proxy. Second, the productivity assumptions in the quantity and price approach are more modest than in the latter. In this case, while facility cost inclusion tends to make estimates obtained with the quantity and price approach lower, the difference in productivity assumptions has the opposite effect. Hence, the overall quantity and price estimates are similar to those obtained from the validation exercise.

Limitations of the laboratory sample processing estimates include their reliance on expert opinion as opposed to directly observed data in each country of interest. Second, the estimates depend on a particular set of technologies being used. For example, automation of slide reading for cervical cytology would introduce larger equipment and supply costs but would also reduce staff costs and change the capacity of the laboratory. Such a change would require a revised assessment of sample processing costs. Finally, costs of certain inputs were assumed to be equal to international market prices. Were these inputs to be produced locally, their value, as measured by their opportunity cost, could be different.

In areas where population density was lowest and paved road networks were scarcest, our estimates of laboratory sample transport costs were highest. Because the laboratory units we considered for processing cervical cytology samples were larger than those used to process HPV DNA samples, the per-sample cost of transport was lower for cervical cytology laboratories. Since major resource inputs such as gasoline and vehicle depreciation were internationally traded goods, their relative costs in different countries had less impact on cost estimates than did the density of road networks and the rural population. Plausible bounds on laboratory sample transport costs were constructed by assuming resources were arbitrarily divisible – that their remainders could be used efficiently for other purposes – or that resources had to be consumed in whole quantities – that their remainders could not be used efficiently for other purposes.

Limitations of the laboratory sample transport estimates include a reliance on national averages for road network density and rural population density. Additionally, changes in elevation and natural obstacles such as the Peruvian Andes affect the estimates of transport distance and time in important ways. While refinement of estimates through the use of provincial data and geographic information system (GIS) data may be desirable, a trade-off exists between the accuracy of estimates and the ability to form comparable estimates for multiple developing countries without additional costly data gathering efforts. Because of this lack of data, the method's predictions have yet to be validated against real-world costs incurred by the operation of a laboratory transport system in a developing country setting.

While the method assumes each laboratory unit is serviced by one transport unit, existing infrastructure or administrative considerations may make laboratory aggregation into larger centers more appealing. While this may lead to economies of scale for laboratory processing costs, laboratory sample transport costs would require new estimates. Similarly, while our estimation method applies to transport of laboratory samples from fixed health clinics to a centralized laboratory, some have proposed the use of mobile screening clinics [44,45]. Because mobile units transport personnel and supplies to the site of care and carry back collected samples to centralized laboratories, efficiencies may be realized in the overall cost of transport. While we make no explicit assumptions about other simultaneously valuable services that laboratory transport could provide such as the transport of blood samples or personnel, such additional services would tend to reduce laboratory sample transport costs. While this may indeed be valuable, mobile clinics also face the challenges related to not being in all locations at all times and thus may face greater difficulty maintaining continuity of care and low levels of loss to follow-up.

When informal and unpaid labor was included in the cost of patient time, the estimate increased substantially. Because cervical cancer screening modalities other than single visit "see and treat" options require multiple trips to clinical sites and potentially district hospitals, the value of patient time traveling, waiting, and receiving care can be substantial. This is the case because even though the per-hour time cost is relatively low, sparse health and transportation infrastructures require travel over long distances and substantial waiting times at health facilities before receiving care.

Limitations of our approach also include a reliance on minimum wage scales to value unpaid and informal labor. Minimum wages can be sector-specific and can overstate the market value of the labor they compensate. Additionally, we used the minimum wage for all types of unpaid and informal labor and did not differentiate high value from low value activities. Finally, the proportion of time spent in the formal and informal economic sectors was not age-specific. Thus, if the target screening population – women from 35–40 – had a different pattern of employment our estimate would not capture the difference.

There is little prior literature on cost estimates for laboratory sample processing in developing country settings. A study of the cost-effectiveness of cervical cancer screening in South Africa used information on laboratory costs from the existing cervical cytology services in the country and included additional test kit costs for HPV DNA testing derived from the manufacturer [21]. A similar study in Thailand provided cost estimates based on information from the government of Thailand for various services including laboratory processing but did not specify how these estimates were derived for services like HPV DNA testing that are not widely available in Thailand at present [23]. A study of the costs and implementation of cervical cytology in Vietnam provided primary data based estimates on cervical cytology laboratory processing but did not include sample transport nor did the data used reflect a screening program with full national coverage [46]. A study of the cost effectiveness of cytology-based screening in Hong Kong reported costs including laboratory services based on data from public and private payers [47]. A study of the cost-effectiveness of cervical cancer screening in Eastern European countries cited lack of data in these countries and instead relied on cost estimates from studies undertaken in the UK [48]. A study of the implementation of government financed, cervical cancer screening in Brazil reports that the results are cost-effective but only describes the effectiveness that the program has achieved [49].

No prior studies were found that directly estimated the cost of laboratory sample transport in developing countries. The method we have employed makes a number of simplifying assumption about population distribution, clinic distribution, and topography. With more complete data, our method could be extended to accommodate these details. Depending on the nature of the additional data, Operations Research techniques for locating new facilities given a set of constraints including maximizing coverage and minimizing cost or distance that have been developed for a variety of other applications could be applied [34]. Other work conducted by the World Bank and the International Center for Tropical Agriculture have evaluated rural transportation infrastructures in developing countries [36,50]. These and similar studies address access to roads, road quality, and speed of travel on roads which may be combined with geographic data to produce estimates of transport costs.

The topic of valuing productive labor, especially of women, in the informal and unpaid sector of the economy is particularly important, especially in countries where less than half of female productive labor takes place in the formal sector. Formalized methods do exist for estimating the value of these activities [51-53]. These methods are designed to include the value of productive labor in estimates of Gross Domestic Product and typically rely on population sampling and use of activity diaries to provide detailed estimates of the quantity of time spent on different activities both paid and unpaid. The value of time spent on unpaid labor is then estimated by such methods as ascribing formal sector wages to unpaid time (opportunity cost approaches) or by using the price paid in the labor market to have another person perform either the mix of unpaid tasks or each individual task separately (market wage approaches). As such, data requirements for these approaches is quite high, and only limited use has been made of these techniques in developing countries and rural areas where the women's formal sector labor participation is often at its lowest.

Large-scale efforts to use cost-effectiveness analysis to assess multiple interventions for many different disease areas have been published relatively recently [25,54,55]. The overriding goal of these efforts has been to inform public policy decisions about the best investments in health, and to contribute to discussions about allocation of public funds. As such, these efforts have focused on providing broad insight by assessing costs and benefits of alternative investments in the context of 14 world regions defined both geographically and in terms of mortality. Although the general methodology is similar, our focus in this analysis is somewhat different in that we are using CEA to evaluate the *technical efficiency *of different cervical cancer screening strategies for reducing mortality from one disease. Our purpose is to provide information to country-based decision makers choosing amongst a range of options for cervical cancer screening. For this goal, it is important to use country-specific data to the extent possible and to explicitly represent cost differences between countries that might make one strategy versus another a more or less attractive locally (e.g., patient transport costs and time). Accordingly, we developed methods to deal with imperfect data availability, generating country-specific cost estimates that included important patient time and infrastructure costs that went beyond simple assay cost estimates.

## Conclusion

When several viable interventions exist for addressing a serious public health problem, CEA can provide one useful type of information for decision makers. When primary cost data are lacking because a specific type of program has yet to be implemented in a given country, it is possible to use other techniques whose simplifying assumptions allow their data requirements to be satisfied with publicly available data. Because of the uncertainty introduced by the simplifying assumptions, the techniques can also be used to generate plausible ranges of estimates for sensitivity analyses. In the context of cervical cancer screening and prevention, use of these techniques helped to quantify important component costs that influenced the overall results of our cost-effectiveness analysis in five developing countries.

## Abbreviations

CEA – Cost-effectiveness analysis

DNA – Deoxyribonucleic acid

GDP – Gross Domestic Product

GIS – Geographic information system

HPV – Human papillomavirus

I$ – International Dollar

PPP – Purchasing Power Parity

UK – United Kingdoms

US – United States

WHO – World Health Organization

WHO-CHOICE – World Health Organization – Choosing Interventions That Are Cost-Effective

## Competing interests

The author(s) declare that they have no competing interests.

## Authors' contributions

JDG participated in the conception and design, acquisition of data, and development of methods for the paper as well as drafting the initial manuscript. SJG participated in the conception and design, development of methods, and in the critical revision of the manuscript for important intellectual content. Both authors read and approved the final manuscript.
